# A new leaf pubescence gene, Hl1th , introgressed into bread wheat from Thinopyrum ponticum and its phenotypic manifestation under homoeologous chromosomal substitutions

**DOI:** 10.18699/vjgb-24-67

**Published:** 2024-10

**Authors:** A.V. Simonov, E.I. Gordeeva, M.A. Genaev, W. Li, I.O. Bulatov, T.A. Pshenichnikova

**Affiliations:** Institute of Cytology and Genetics of the Siberian Branch of the Russian Academy of Sciences, Novosibirsk, Russia; Institute of Cytology and Genetics of the Siberian Branch of the Russian Academy of Sciences, Novosibirsk, Russia; Institute of Cytology and Genetics of the Siberian Branch of the Russian Academy of Sciences, Novosibirsk, Russia Novosibirsk State University, Novosibirsk, Russia; Institute of Cytology and Genetics of the Siberian Branch of the Russian Academy of Sciences, Novosibirsk, Russia Novosibirsk State University, Novosibirsk, Russia; Institute of Cytology and Genetics of the Siberian Branch of the Russian Academy of Sciences, Novosibirsk, Russia Novosibirsk State Agrarian University, Novosibirsk, Russia; Institute of Cytology and Genetics of the Siberian Branch of the Russian Academy of Sciences, Novosibirsk, Russia

**Keywords:** trichome, digital characteristics of pubescence, phenotypic markers, microsatellite markers, interactions of genes, трихомы, цифровые характеристики опушения, фенотипические маркеры, микросателлитные маркеры, взаимодействие генов

## Abstract

Blue-grain lines were created on the basis of the spring bread wheat variety Saratovskaya 29 (S29) with chromosome 4B or 4D replaced with chromosome 4Th from Thinopyrum ponticum. The leaf pubescence of the two lines differs from S29 and from each other. In this work, we studied the effect of these substitutions on the manifestation of this trait. To quantify pubescence, the LHDetect2 program was used to determine trichome length and number on the leaf fold microphotographs. The key gene Hl1 on chromosome 4B and another unidentified gene with a weak effect determine the leaf pubescence of the recipient S29. Their interaction leads to the formation of trichomes of up to 300 microns in length. Replacement of both copies of chromosome 4B with two copies of wheatgrass chromosome 4Th modifies leaf pubescence in line S29_4Th(4B) so that the leaf pubescence characteristic of S29 becomes more sparse, and trichomes of up to 600– 700 μm in length are formed. Additionally, we described modification of pubescence in the substitution line S29_4Th(4D) where chromosome 4D that does not carry any pubescence gene was replaced. Under this substitution, trichomes of up to 400 μm in length were formed and the average length of trichomes on the underside of the leaf was reduced. The replacement of the Hl1 gene in the lines was also confirmed by the allelic state of the linked microsatellite marker Xgwm538. Thus, as a result of the studies, a new leaf pubescence gene introgressed from Th. ponticum into bread wheat was identified. We designated it as Hl1th. For the purpose of selection, we propose to use the unlicensed informative microsatellite markers Xgwm538 and Xgwm165, allowing chromosomes 4A, 4B, 4D and 4Th to be distinguished.

## Abstract (ru)

На основе сорта яровой мягкой пшеницы Саратовская 29 (С29) были созданы голубозерные линии C29_4Th(4B) и C29_4Th(4D) с соответствующим замещением хромосом 4B и 4D хромосомой 4Th от пырея вида Thinopyrum ponticum. У этих линий опушение листа отличается от реципиента и различается между собой, в связи с чем нами проведено исследование эффекта замещений на проявление данного признака. Для количественной оценки опушения была применена программа LHDetect2, определяющая длину и число трихом на микрофотографиях. Опушение листа у сорта С29 определяется главным геном Hl1 в хромосоме 4B и еще одним геном со слабым эффектом с неизвестной хромосомной локализацией. Их взаимодействие приводит к формированию трихом длиной до 300 мкм. Замещение пары хромосом 4B на пару хромосом 4Th пырея модифицирует опушение листа у линии C29_4Th(4B). Характерное для сорта С29 опушение листа у линии C29_4Th(4B) становится реже, при этом образуются трихомы длиной до 600–700 мкм. Замещение гена Hl1 на Hl1th у линии C29_4Th(4B) также подтверждается аллельным состоянием сцепленного с геном Hl1 микросателлитного маркера Xgwm538. Нами была описана модификация опушения у замещенной линии C29_4Th(4D), где произошло замещение пары хромосом 4D, не содержащей гена опушения. Экспрессирующиеся совместно гены Hl1 и Hl1th у линии C29_4Th(4D) в хромосомах 4B и 4Th соответственно, формируют трихомы длиной более 400 мкм. Однако в таком генотипе снижается средняя длина трихом в сравнении с реципиентом. Таким образом, в результате проведенных исследований идентифицирован новый ген опушения листа, интрогрессированный из вида Th. ponticum в мягкую пшеницу, который мы обозначили как Hl1th. Для ведения отбора мы предлагаем использовать находящиеся в открытом доступе информативные микросателлитные маркеры Xgwm538 и Xgwm165, позволяющие различать хромосомы 4A, 4B, 4D и 4Th.

## Introduction

Alien hybridization is widely used in breeding programs to transfer new useful traits into bread wheat (Triticum aestivum, AABBDD, 2n = 6x = 42). For this purpose, both closely related species from the genus Triticum L. with similar genomes, such as Aegilops, are used, and species from other genera of the family Poaceae. Decaploid wheatgrass species Thinopyrum ponticum (Podp.) Barkworth & D.R. Dewey (2n = 10x = 70, StStStStEeEeEbEbExEx syn. Agropyron elongatum Host., Elytrigia pontica (Podp.) Holub) belongs to the tertiary gene pool of wheat relatives, and since the mid-20th century it has served as a source of useful genes in wheat breeding (Kroupin et al., 2019). With its tolerance to biotic and abiotic stress factors, Th. ponticum has become a donor of effective genes for resistance to various wheat diseases: root rot, leaf, stem and stripe rust, powdery mildew (Li H. et al., 2004; Li H., Wang, 2009; Niu et al., 2014; Wang et al., 2019; Li M. et al., 2021; Yang et al., 2023).

For 30 years, the Institute of Cytology and Genetics SB RAS has been expanding the collection of substituted, isogenic and alloplasmic lines of bread wheat based on the spring variety Saratovskaya 29 (S29) and other varieties. They carry either individual chromosomes, or certain rearrangements in the wheat chromosomes, or the cytoplasm of related species acquired through alien hybridization. Many of these introgressions have been identified using cytological or molecular methods (Leonova et al., 2008; Adonina et al., 2021; Shchukina et al., 2022; Pershina et al., 2023). The 4Th chromosome pair of the species Th. ponticum was transferred into the genome of S29 from the winter wheat variety Meropa developed in Bulgaria (Gordeeva et al., 2019). As a result, a substitution line with blue anthocyanin grain color was obtained. It has been established that the Ba gene responsible for the blue color of the aleurone layer (Blue aleuron) is located on chromosome 4Th of wheatgrass Th. ponticum (Zeven, 1991).

Using GISH analysis, it was shown that the centromeric and pericentromeric regions of chromosome 4Th originate from the E-genome chromosome, and the distal regions of its two arms, from the St-genome chromosome (Zheng et al., 2006). After the selection of hybrid plants in the generation BC7F2-3, according to the results of cytological and molecular analyses, no recombination was found between the wheat and wheatgrass chromosomes. Therefore, a complete replacement of 4B or 4D chromosome pair with 4Th chromosome pair occurred (Gordeeva et al., 2022). In addition to the blue color of the grain, the changes in the pubescence of leaf blades were visually and tactilely detected in comparison with the recipient in the obtained substitution lines S29_4Th(4B) and S29_4Th(4D) (Gordeeva et al., 2022).

Leaf pubescence is known to be an adaptive trait (Kaur, Kariyat, 2020). Hairiness in rice affects transpiration and drought tolerance, thereby increasing the yield (Hamaoka et al., 2017). A positive effect of this trait on photosynthetic parameters of wheat plants under drought conditions has been shown (Pshenichnikova et al., 2019; Osipova et al., 2020). The pubescence of cereal leaves is presented as outgrowths of epidermal cells – non-secretory trichomes; their length and density varies greatly among the carriers of different genomes (Pshenichnikova et al., 2017). For example, for winter wheat cultivars, leaf pubescence is not typical (our unpublished data), but the phenotypic diversity for this trait among spring wheat cultivars may depend on the region where they were developed (Genaev et al., 2012a).

The occurrence of this trait among cereals corresponds to the “law of homological series”, which was formulated in 1920 by N.I. Vavilov (Vavilov, 1935). Among the cereal species, such as rye, barley, rice, and other, more distant species, the accessions may be found with leaf pubescence similar to that found in wheat (Shvachko et al., 2020). The main dominant gene Hl1 of cv. S29 is located on chromosome 4BL and is responsible for the formation of medium-length trichomes (Maistrenko, 1976; Dobrovolskaya et al., 2007). The non-localized minor gene Hl3 is also known to form small trichomes and slightly enhances the effect of the Hl1 gene (Maistrenko, 1976). In the diploid genome of barley (Hordeum vulgare L.), the genes for leaf blade and for leaf sheath pubescence were mapped on the long arms of chromosomes 3H and 4H, respectively (Saade et al., 2017; Shvachko et al., 2020). In synthetic hexaploid wheat, the leaf sheath and leaf margin pubescence was associated with Aegilops tauschii Coss genome and the responsible gene was found in the long arm of 4D chromosome (Dobrovolskaya et al., 2007; Wan et al., 2015)

The present work is aimed at studying the phenotypic manifestation of a new allele of the Hl1 gene for leaf pubescence transferred with chromosome 4Th from the species Thinopyrum ponticum to the genome of wheat cultivar S29. At the same time, work was carried out to identify the substitution of chromosomes 4B or 4D with chromosome 4Th of wheatgrass using molecular markers. The aim of the work was to study the phenotypical interaction between two genes during the replacement of chromosomes 4B and 4D by quantifying the length and number of trichomes.

## Materials and methods

The plant material was represented by the spring recipient cultivar Saratovskaya 29 (S29) and two single chromosome substitution lines S29_4Th(4B) and S29_4Th(4D) (other previously used synonyms, respectively: s:S29_4Th(4B) and s:S29_4Th(4D), Gordeeva et al., 2019, 2022). According to cytological and molecular data (Gordeeva et al., 2019, 2022) the substitution lines are stable.

Analysis of leaf fold image. Microphotographs of transverse folds on the upper and lower sides of a boot leaf were used to determine the number and length of trichomes according to the protocol developed at the Institute of Cytology and Genetics SB RAS (Doroshkov et al., 2009). Images were obtained at the Center for Microscopic Analysis of the Institute of Cytology and Genetics SB RAS on a Carl Zeiss Axioscop 2 plus microscope through a 5x/0.12 lens. The microscope was equipped with an AxoCam HRc digital camera with a TV2/3C 0.63x adapter. The physical size of the field of view during shooting was 2730 × 2163 μm, the resolution of digital photography was 1300 × 1030 pixels. The physical pixel size was 2.1 microns. To obtain digital characteristics of leaf pubescence, the images were analyzed using the computing resources of the Bioinformatics Center for Common Use using the LHDetect2 program developed at the Laboratory of Evolutionary Bioinformatics and Theoretical Genetics of the Institute of Cytology and Genetics SB RAS (Genaev et al., 2012b). The program identifies trichomes, determines their length and produces the result as a sequence of numbers in a text file.

For each of two plants of the same genotype, 12 microphotographs were analyzed, with six folds from the upper and lower sides of the boot leaves. Trichomes formed under the influence of different genes within each class differ greatly in length. Therefore, the length values were presented in logarithmic scale. The calculation of the average length was carried out both in absolute values (microns) and as a decimal logarithm. Additionally, the distribution of trichome lengths and numbers was analyzed.

Statistical processing. The significance of differences between genotypes in length and number of trichomes was assessed using Student’s t-test, for which MS Excel with the statistical add-in AgCStat was used (Gonchar-Zaykin, Chertov, 2003). The criterion for the significance of differences p < 0.05, 0.01 and 0.001 was indicated by one, two and three symbols, respectively: a – the difference between the substitution line and the recipient, b – the difference between the two substitution lines, * – the difference between the upper and lower sides of the leaf within the genotype. Diagrams of trichome length distribution were constructed in PAST v.3.0 statistical package. The data on the trichomes recognized from a total of six images were used for each genotype

Genotyping. DNA was isolated from young leaves according to J. Plaschke et al. (1995). Samples diagnostic was made using PCR with microsatellite markers (SSR, simple sequence repeats) developed for chromosomes of the fourth homoeologous group according to recommended amplification programs (Röder et al., 1998). For this purpose, the markers Xgwm538 and Xgwm165 were chosen.

The Xgwm538 marker is located on the long arm of chromosome 4B approximately 2.1 cM proximal to the Hl1 gene in wheat (Dobrovolskaya et al., 2007). It showed amplification products of 157 bp in size for cv. S29 genome, and 155 bp for cv. Purple Feed (Dobrovolskaya et al., 2007). For cv Chinese Spring, it showed three fragments of 137, 147 and 152 bp, with the last product corresponding to chromosome 4B, and the others amplified from chromosome 4D as shown in nulltetrasomic lines (Brooks et al., 2006). This marker is often used, for example, to map the genes for infection resistance (Sukhwinder-Singh et al., 2003; Brooks et al., 2006; Singh et al., 2012). In our work, we used classical primers of the Xgwm538 marker (Röder et al., 1998), which show PCR products from chromosomes 4B and 4D. This marker also showed multiple polymorphisms in two species of wheatgrass and wheat-wheatgrass hybrids (Kroupin, 2011). The Xgwm165 marker is located on the long arms of chromosomes 4B and 4D and on the short arm of chromosome 4A with pericentric inversion (Röder et al., 1998). It is often used to map different genes and QTLs (Pshenichnikova et al., 2012; Salem, Mattar, 2014; Shchukina et al., 2018).

For PCR, a ready-made mixture of BioMaster HS-Taq reagents from BiolabMix LLC was used. PCR products were separated by electrophoresis in 3.5 % agarose gel with the addition of ethidium bromide. For electrophoresis, TBE buffer (Tris-borate-EDTA) and DNA fragment length marker Step50+ (BiolabMix, Novosibirsk, Russia) were used.

Growing conditions. The plant material was grown in a hydroponic greenhouse at the Center for Collective Use of Plant Reproduction of the Institute of Cytology and Genetics SB RAS. Growing conditions: lighting with 600W HPS lamps with adjustable suspension height (up to 45–50,000 lux at the level of the upper leaves) for 12–14 hours at a temperature of 18–20 °C at night and 24–26 °C during the day. Soil substrate: expanded clay, moistened with Knop nutrient solution three times a day.

## Results

**Fig. 1.**
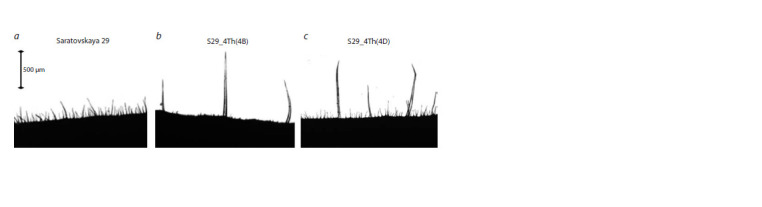
Effect of substitution of chromosomes 4B and 4D with chromosome 4Th on the leaf pubescence phenotype in S29. The photographs show trichomes from the folds of the upper part of the leaf in transmitted light.

The pubescence of line S29_4Th(4B) with the replacement of chromosome 4B by chromosome 4Th tactilely distinguished it from the recipient. According to the results of microscopic observations and a detailed study of the leaf pubescence morphology (the method described above), both substitution lines, S29_4Th(4B) and S29_4Th(4D), differed from S29 and from each other. Figure 1 shows microphotographs of leaf folds of the three genotypes, which demonstrate visually distinguished trichomes of different lengths. Digital processing of microphotographs of S29 leaves showed that the average length of trichomes was 64.5 μm on the underside of the leaf and 67.1 μm on the top (see the Table). Their maximum length did not exceed 306 μm. Despite the fact that the longest trichomes were formed on the upper side of the leaf, the density of pubescence and the sum of the lengths of all trichomes on the lower side of the leaf were one and a half times higher than on the upper side (see the Table).

**Table 1.**
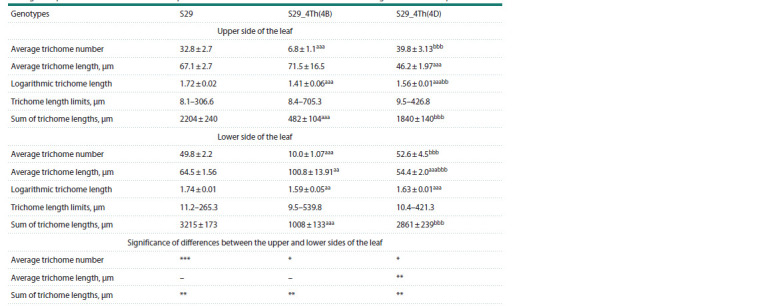
Average morphometric characteristics of leaf pubescence of cv. S29 and substitution lines with introgression from Th. ponticum Notе. Values marked with superscript “a” are significantly different between the substitution lines and the recipient S29; values marked with superscript “b” are significantly different among the substitution lines; values with superscript asterisks “*” are significantly different between the leaf sides in the same genotype; numbers of superscript symbols indicate significant levels: p < 0.05ab*, p <0.01aabb**, p < 0.001aaabbb***.

The number of trichomes in line S29_4Th(4B) was reduced fivefold in comparison with the recipient (see the Table). On the upper side, single trichomes up to 705.3 μm long were observed, and on the lower side – up to 539.8 μm, which was two times greater than the maximum length of trichomes in S29. Trichomes were more common on the underside of the leaf than on the upper side, and the sum of lengths was twice the sum of the lengths of trichomes from the upper side. The average length of the trichomes on the upper side of the leaf in the line and in S29 did not differ significantly; however, on the lower side of the leaf, the difference in the average lengths of the trichomes was significant. The sum of the lengths of trichomes on both leaf sides in line S29_4Th(4B) was significantly reduced compared to S29.

**Fig. 2.**
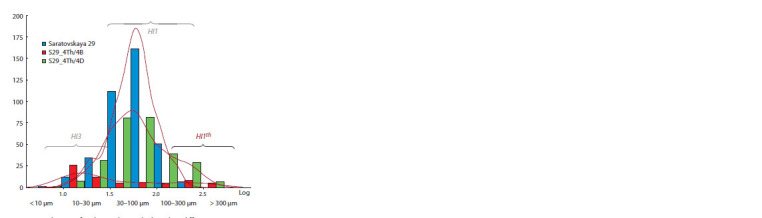
Distribution of trichome density by length in different genotypes. The X-axis scale is logarithmic. The Y-axis scale presents the number of trichomes by class from six images with a total width of approximately 13 mm (the width of one image is 2.163 mm). The data is presented for the lower surface of the leaf.

Small and large trichomes differ in length greatly on the leaf fold of line S29_4Th(4B) (Fig. 1b). Figure 2 shows the distribution of trichomes of different lengths according to their number. In line S29_4Th(4B) (red bars), the number of trichomes with a length from 30 to 300 μm is significantly reduced, but a class of trichomes with a length of more than 300 μm has appeared, which is absent in S29. The difference in the average trichome logarithmic lengths of line S29_4Th(4B) and the recipient was significant on both sides of the leaf (see the Table). The sum of the trichome lengths of the substitution line is also 3–4 times smaller on both sides compared to S29.

A different morphology of trichomes was observed on microphotographs of leaves in line S29_4Th(4D). The dense canopy of pubescence of S29 is preserved, but additional longer trichomes have been formed. Tactilely, they were hardly noticeable against the general background, but in microphotographs they stood out above the main trichome layer typical of S29 (Fig. 1b). The maximum trichome length in line S29_4Th(4D) exceeded 400 microns on both sides of the leaf, whereas in S29 it was slightly more than 300 microns on the upper side and more than 200 microns on the lower. In comparison with S29, the total number of trichomes increased insignificantly. At the same time, the difference in this indicator between lines S29_4Th(4D) and S29_4Th(4B) was significant on both sides of the leaf in favor of the first line. The sum of the trichome lengths in line S29_4Th(4D) decreased slightly compared to S29, but it was 3–4 times higher than that of line S29_4Th(4B). Nevertheless, the line with S29_4Th(4D) had a significantly lower average trichome length than the recipient variety with S29. In terms of the average logarithm length of trichomes, line S29_4Th(4D) differed from both S29 and line S29_4Th(4B). In Figure 2, the distribution of trichomes in line S29_4Th(4D) clearly demonstrates a multiple decrease in the number of trichomes of average length and the presence of a class of trichomes longer than in S29.

**Fig. 3.**
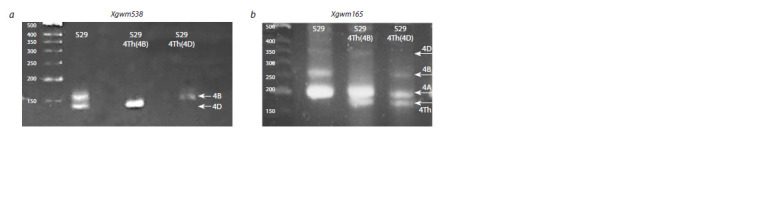
Electrophoregram of PCR products obtained as a result of DNA amplification of S29 and substitution lines S29_4Th(4B) and S29_4Th(4D) using microsatellite markers Xgwm538 (a) and Xgwm165 (b).

The microsatellite marker Xgwm538 amplifying a 157-bp product is closely linked to the Hl1 gene on chromosome 4B in S29. Using Chinese Spring nulli-tetrasomic lines, it was shown that this marker amplifies a fragment 174 bp in size, specific for chromosome 4B, and two fragments (147 and 137 bp) for chromosome 4D. In S29, only one product less than 150 bp is detected, corresponding to chromosome 4D. The Xgwm538 marker confirmed in our work the presence of chromosomal substitution in the genome of S29 in both substitution lines (Fig. 3a). Line S29_4Th(4B) lacked a fragment larger than 150 bp, which corresponds to the diagnostic fragment for chromosome 4B. On the contrary, line S29_4Th(4D) did not have a fragment smaller than 150 bp, which indicates the presence of chromosome 4D. Thus, the polymorphic marker Xgwm538 detects wheat chromosomes 4B and 4D of S29 and is not amplified on wheatgrass chromosome 4Th.

The microsatellite marker Xgwm165 used in our work amplifies fragments on chromosomes 4A, 4B and 4D. We detected bright signals of amplification products of this marker for S29 in an agarose gel (Fig. 3b) with sizes of ~200, ~260 bp, as well as a less pronounced signal with a size of ~350 bp.

A PCR fragment about 200 bp was also observed in both substitution lines, which corresponds to chromosome 4A. Line S29_4Th(4B) lacked a 260 bp PCR product, but a 180 bp fragment was detected. A fragment of the same length (180 bp) was detected in line S29_4Th(4D) in combination with PCR products 200 and 260 bp in size as in S29, but there was no signal of 350 bp (Fig. 3b). PCR results obtained using Xgwm165 suggest that 180 bp fragment is synthesized from chromosome 4Th and therefore can be used in determining this chromosomal substitution.

## Discussion

The first identified wheat leaf pubescence gene with established chromosomal localization was the Hl1 gene on chromosome 4B of cv. S29 (Maistrenko, 1976). The replacement of chromosome 4B of this variety with the chromosome of the non-pubescent cultivar Yanetzkis Probat changes the morphology of pubescence. This genotype has a noticeably reduced number of trichomes as well as their size (Doroshkov et al., 2016). This phenotype is determined by the presence of gene Hl3 with a weak effect. In the absence of Hl1 and Hl3, trichomes are practically not formed on the leaves of the S29 isogenic line, as was shown by the development of a glabrous isogenic line of this cultivar (Doroshkov et al., 2016).

Long, sparse trichomes are not typical for leaves of S29. Their appearance in the two substitution lines is apparently determined by a new variant of the pubescence gene transferred from Th. ponticum. Our studies indicate that wheatgrass chromosome 4Th carries a new allelic variant of the gene, orthologous to the wheat Hl1 gene, but with a different phenotypic manifestation. The wheatgrass gene, which replaced wheat gene Hl1 in line S29_4Th(4B), or was added to it in line S29_4Th(4D), not only forms long trichomes, but also reduces their total number. In accordance with the rules of the Catalog of Gene Symbols for Wheat (McIntosh et al., 2013), we designated the new allele with the symbol Hl1Th. Previously, the leaf margin pubescence gene Hsh (otherwise Hs) was found on chromosome 4H of barley (Hordeum vulgare L.) in a region comparable with chromosomes 4B and 4D (Korzun et al., 1999). A QTL associated with leaf margin pubescence was identified on chromosome 4D using the ITMI mapping population (Dobrovolskaya et al., 2007). In this work, we supplemented the homologous series of pubescence genes for the fourth group of chromosomes of cereal plants.

Previously, we suggested that the Hl1 gene is responsible for the number of trichomes on the leaf surface, that is, for pubescence density (Doroshkov et al., 2014). In the substitution line C29_4Th(4B), the wheatgrass gene Hl1th in the absence of Hl1 stimulated the formation of single long trichomes. When combined in one genotype in line S29_4Th(4D), the Hl1th gene apparently has a suppressive effect on Hl1, reducing the average length of trichomes and the sum of their lengths. Genes promoting formation of long, rare trichomes on the leaf surface were also localized on other chromosomes of different cultivars. The Hl2 gene located chromosome on 7B, was found in the Chinese cultivar Hong-mang-mai (Taketa et al., 2002). The Hl2aesp gene was introgressed from chromosome 7S of Aegilops speltoides Taush. in сv. Rodina (Pshenichnikova et al., 2007). In the species Triticum timopheevii, the Hltt gene with a similar phenotypic manifestation was found on chro-mosome 5A (Simonov et al., 2021).

The substitution lines of S29 were obtained to study the genes regulating anthocyanin biosynthesis. Wheatgrass chromosome 4Th carries the Ba gene responsible for the blue color of the grain aleurone layer (Gordeeva et al., 2019), which is also a phenotypic marker of the presence of this chromosome in the genome. However, the grain color is manifested both during the replacement of chromosome 4B and chromoso-me 4D. The phenotypic effect of introgressed pubescence can serve as a morphological marker for plant selection when obtaining the blue-grained forms with a certain chromosomal substitution in cultivars having the S29-like type of pubescence

In this work, we studied polymorphism in microsatellite markers that were previously associated with chromosomes of the 4th group and with the Hl1 gene on chromosome 4B in particular (Dobrovolskaya et al., 2007). Figure 3a shows the Xgwm538 marker, which is located near the Hl1 gene; in S29, it showed a 157 bp fragment (Dobrovolskaya et al., 2007). This marker can clearly indicate which of the wheat chromosomes, 4B or 4D, is replaced by 4Th from Th. ponticum. It was previously noted that the Xgwm538 marker demonstrates specific fragments for the genomes of Th. intermedium and Th. elongatum (Kroupin et al., 2011). But in our work, no signals were detected from chromosome 4Th consisting of fragments of the St and E genomes of Th. ponticum (Zheng et al., 2006).

Since the wheatgrass chromosome 4Th does not recombine with homeologs, the Xgwm165 marker was used in this work, amplifying products specific to chromosomes 4A, 4B and 4D (Röder et al., 1998). According to various molecular maps of the GrainGenes database (https://graingenes.org/cgi-bin/ GG3/browse.cgi), on chromosome 4B this marker is located proximal to Xgwm538 at a distance of about 20–30 cM. In the genome of S29, this marker synthesized different PCR products (Fig. 3b) for chromosomes of the 4th homoeologous group: for 4A, about 200 bp, for 4B, about 260 bp, and a weak signal of about 350 bp, presumably for chromosome 4D. Xgwm165 also exhibited a 180 bp fragment for chromosome 4Th. This makes it possible to differentiate wheat plants with different chromosomal substitutions within the fourth group if the parent varieties are not characterized by S29 pubescence.

Trichomes form a special microclimate on the leaf surface; they are able to influence the stability of the surface air layer, changing laminar flows to turbulent ones (Schreuder et al., 2001). Turbulent flows, in turn, contribute to more dynamic gas exchange. Accordingly, changes in the parameters of surface pubescence should affect the parameters of stomatal conductance, the absorption of carbon dioxide and the intensity of moisture evaporation. In the future, it is planned to study these lines on the dynamics of photosynthetic parameters under various growing conditions, in particular, during adaptation to drought.

## Conclusion

In our work, for the first time, a new allelic variant of the leaf pubescence gene Hl1th transferred from the decaploid species Thinopyrum ponticum to bread wheat was discovered and described using digital phenotyping. Its observed phenotypic manifestation against the background of the wheat genome was significantly different from the effect of the wheat gene Hl1. In its morphology, it is similar to that of the genes Hltt and Hl2aesp localized in chromosomes 5A and 7S of the related cereals T. timopheevii and Ae. speltoides. The created lines make it possible to compare the adaptive value of similar leaf pubescence morphotypes controlled by different genes within the same model recipient genotype.

## Conflict of interest

The authors declare no conflict of interest.
